# Decreasing prevalence and time trend of gastroschisis in 14 cities of Liaoning Province: 2006–2015

**DOI:** 10.1038/srep33333

**Published:** 2016-09-14

**Authors:** Na Li, Yan-Ling Chen, Jing Li, Li-Li Li, Cheng-Zhi Jiang, Chen Zhou, Cai-Xia Liu, Da Li, Ting-Ting Gong, Qi-Jun Wu, Yan-Hong Huang

**Affiliations:** 1 Department of Obstetrics and Gynecology, Shengjing Hospital of China Medical University, Shenyang, China; 2 Liaoning Women and Children’s Health Hospital, Shenyang, China; 3 Department of science and education, Shenyang Women and Children Health Care Centre, Shenyang, China; 4 Department of children’s health prevention, Shenyang Women and Children Health Care Centre, Shenyang, China; 5 School of Environmental and Chemical Engineering, Shenyang Ligong University, Shenyang, China; 6 Department of information statistics, Shenyang Women and Children Health Care Centre, Shenyang, China; 7 Department of Clinical Epidemiology, Shengjing Hospital of China Medical University, Shenyang, China

## Abstract

To identify trends in the prevalence of gastroschisis on the basis of a large population-based observation study with cases identified by the Liaoning Birth Defects Registry including 14 cities over the course of a 10-year period. Data were obtained from the aforementioned registry which was maintained by the Liaoning Women and Children’s Health Hospital, a comprehensive care institution as well as being responsible for the women’s and children’s health care guidance in this province. Gastroschisis prevalence, percent change, annual percent change (APC), and contribution rates of each city were calculated. We observed 747 cases of gastroschisis among 3,248,954 live births, for a prevalence of 2.30 per 10,000 births. The gastroschisis prevalence significantly decreased by 12.63% per year in Liaoning Province. Although the decreasing trends were observed in all these 14 cities, significant results were only observed in Shenyang (APC = −16.31%), Tieling (APC = −20.23%), and Chaoyang (APC = −13.50%). Notably, Tieling, Shenyang, and Yingkou were the three major cities which contributed almost 37.17% of the decreasing trend of gastroschisis in Liaoning Province. In conclusion, our findings demonstrate that the prevalence of gastroschisis has been decreasing during the recent decade among 14 cities in Liaoning Province.

Gastroschisis is the evisceration of the fetal intestine through a defect in the paraumbilical anterior abdominal wall with herniation of gastrointestinal structures into the amniotic cavity^1^. Babies born with this condition are more likely to be born prematurely and to have had poor fetal growth^1^. While the prognosis for these babies who receive treatment via surgery are generally favorable, with a greater than 90% survival rate^2^,^3^. In many developed and developing countries, previous studies indicated that there has been two- to fourfold increase in the birth prevalence of gastroschisis from 1980 to 2010^4^. Local and state registries in the United States have shown increases in the prevalence of gastroschisis during this period^2^,^4^,^5^,^6^,^7^,^8^,^9^. For instance, Kirby *et al*.^5^ demonstrated that the prevalence increased from 2.32 per 10,000 live births in 1995 to 4.42 per 10,000 live births in 2005. Furthermore, a recent report from Jones *et al*.^6^ suggested that prevalence has continued to increase beyond 2005 on the basis of data from 14 States. A similar trend has been observed in other regions of the world as well, including Europe and Australia^10^,^11^,^12^,^13^.

However, the studies describing the time trend and prevalence of gastroschisis have been limited in China. Zhu *et al*.^14^ utilized the national monitoring database to report epidemiologic data on gastroschisis from 1996 to 2000. Additionally, Xu *et al*.^15^ found no significant change in the trend and prevalence of gastroschisis from 1996 to 2007. While, these aforementioned databases were collected one or two decades ago. Notably, to the best of knowledge, no study has demonstrated the time trends and prevalence of gastroschisis in China by the data of the recent decade. Whether prevalence of this disease has continued to be constant has been still unknown. As one of the most important provinces in China, Liaoning Province, covers an area of 145,900 square kilometers and has a population of almost 42 million, contributed greatly to the development of China in the past decades. Nevertheless, no formal assessment of this population had been made. Therefore, to address these aforementioned research questions, we examines gastroschisis prevalence among live birth infants in Liaoning Province for the 10-year period from 2006–2015.

## Results

Table 1 presents the number of live births of each city in Liaoning Province during the 10-year observational period. During this period, the overall number of live births was highest in 2014 (364,400) but lowest in 2015 (298,437). Additionally, when compared with cities, Shenyang, the capital city of this province, had the largest number of live births in each year. In contrast, Benxi had the smallest number of live births.

The prevalence of gastroschisis in each city in Liaoning Province is demonstrated in Table 2. During 2006–2015, 747 gastroschisis cases were detected among 3,248,954 live births (prevalence rate = 2.30 per 10,000 live births). Liaoyang (3.58 per 10,000 live births), Fushun (3.40 per 10,000 live births), and Shenyang (3.14 per 10,000 live births) were the top three leading cities in Liaoning Province. In contrast, Yingkou (1.24 per 10,000 live births), Tieling (1.24 per 10,000 live births), and Anshan (1.43 per 10,000 live births) were the three cities with lowest gastroschisis prevalence.

**Figure 1:**
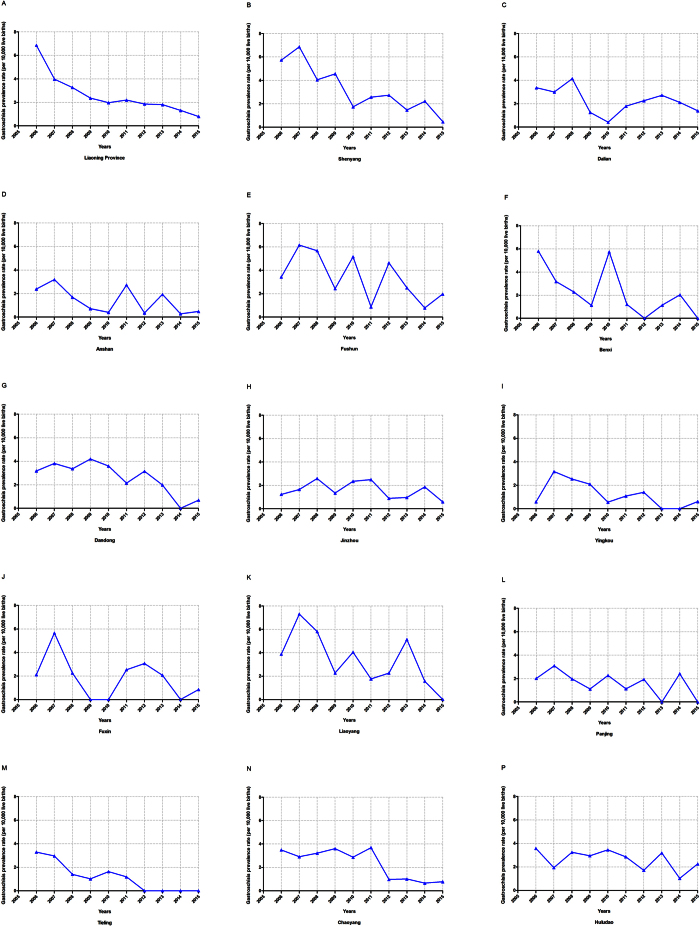
Trends in gastroschisis prevalence (per 10,000 live births) of each city in Liaoning province, 2006–2015. (**A**) Liaoning province; (**B**) Shenyang; (**C**) Dalian; (**D**) Anshan; (**E**) Fushun; (**F**) Benxi; (**G**) Dandong; (**H**) Jinzhou; (**I**) Yingkou; (**J**) Fuxin; (**K**) Liaoyang; (**K**) Panjing; (**L**) Tieling; (**M**) Chaoyang; (**N**) Huludao.

Figure 1 depicts the time trend of gastroschisis prevalence in each city of Liaoning Province during the period of 2006–2015. The overall prevalence significantly decreased by 76.05% from 33.58 to 8.04 per 10,000 live births, or 12.63% per year (Table 3). Among these 14 cities, significant decreasing trends were also observed in three cities, Shenyang (APC = −16.31%), Tieling (APC = −20.23%), Chaoyang (APC = −13.50%). Notably, borderline significant decreasing trend was observed in Dandong, with APC of −8.42% (95%CI: −16.69 to −0.66).

Table 4 presents the contribution rates of each city of overall decreasing trend of Liaoning Province. Tieling, Shenyang, and Yingkou were the three major cities which contributed almost 37.17% of the decreasing trend of gastroschisis prevalence.

## Discussion

To the best of our knowledge, this is the first report describing the time trend of gastroschisis prevalence on the basis of the data from the recent decade in China. Our findings demonstrated prevalence of gastroschisis significantly decreased by 76.05% from 33.58 to 8.04 per 10,000 live births, or 12.63% per year for the 10-year period from 2006 through 2015 in Liaoning Province. Additionally, decreasing trends were observed in all 14 cities of this province.

The overall prevalence of gastroschisis for Liaoning Province in our study during 2006 to 2015 was 2.30 cases per 10,000 live births. This was slightly lower than the reported prevalence in China between 1996 and 2007, which was 2.54 per 10,000 live births^15^. However, compared with the prevalence of gastroschisis (1.6 per 10,000 births) during 1986 to 1987 in China^14^, the prevalence during 2006 to 2015 was significantly higher. Similar patterns were also observed in other countries. For example, on the basis of 25 population-based registries in 15 European countries, Loane *et al*.^10^ reported that the prevalence of gastroschisis during 1980 to 1984 increased from 0.54 (per 10,000 births) to 2.12 (per 10,000 births) during 2000 to 2002. In addition, Williams *et al*. demonstrated that the prevalence of gastroschisis during 1976 to 2000 (2.3 per 10,000 live births) was significantly higher than the prevalence (0.8 per 10,000) through the Metropolitan Atlanta Congenital Defects Program during 1968 to 1975^16^. Similar studies from Norway, Australia, and England also demonstrated statistically significant increasing prevalence as well^11^,^13^,^17^. However, limited studies has estimated the prevalence of gastroschisis during the recent decade which restricted the comparison. From 14 population-based state surveillance programs in the United States, Jones *et al*.^6^ presented that 4,497 gastroschisis cases were detected among 9,264,540 live births (prevalence rate = 4.9 per 10,000 live births) during 2006 to 2012. The prevalence of our study during 2006 to 2012 was relatively lower than the rate of aforementioned study (2.72 per 10,000 live births versus 4.9 per 10,000 live births).

Although the decreasing trends were observed in all 14 cities, significant results were only observed in Shenyang (APC = −16.31%), Tieling (APC = −20.23%), and Chaoyang (APC = −13.50%). Additionally, we observed significant geographical variation in prevalence within Liaoning, with some city (e.g. Yingkou) having half the prevalence compared to Shenyang. This difference could not be attributed to the ascertainment of gastroschisis since all the cases were reviewed and confirmed through a group of state-level experts in medical genetics and pediatrics. Possible different development of these cities could possibly explain the differences. Development of a region may be associated with many environmental exposures including maternal age at delivery^18^,^19^,^20^, socio-economic status^20^,^21^, maternal diet and drug use during pregnancy^20^,^22^,^23^ which were potential risk factors for gastroschisis. Nevertheless, because of the access on the data, we could not test these hypotheses. Herein, future studies are warranted to further investigate these issues.

Our study have several strengthens. First, this report is a population-based observation study describing the time trend of gastroschisis prevalence in all 14 cities of Liaoning Province providing the possibility of comparison between cities. Additionally, this is a relatively longer time period of data as well as accurate results that provided a more recent report on the status of gastroschisis prevalence, with data up to 2015. Of note, previous to the present study, only a few studies have reported the prevalence of gastroschisis in China. Despite the clear strengths of our study, prudence be used when interpreting these findings. First, we have no access to the demographic factors (e.g., maternal age, race/ethnicity) for all live births in Liaoning Province. For example, we could hardly confirm the phenomenon that the prevalence of gastroschisis was especially higher in younger mothers (<20 years). Additionally, although we were unable to access congenital malformation data in Liaoning Province prior to 2006, our report provided the trend of gastroschisis prevalence on the basis of the recent decade which has been very limited in developing countries. Second, the maximal diagnosis time for gastroschisis cases was the seventh day after birth^15^. We did not include gastroschisis cases confirmed after the seventh day which result in slightly lower prevalence of gastroschisis in the present study than in studies that include longer periods for confirmed diagnoses. However, limited number of cases (n = 4) were diagnosed after that time point in this study during the ten-year observation period.

In summary, a decreasing trend of gastroschisis prevalence was observed in Liaoning Province over the last 10 year which was the most recent and detailed evidence for time trends in the prevalence of gastroschisis in China. The present study not only supports the policy which was proposed by the government but helps them to understand the recent dynamics of gastroschisis prevalence. Since several cities still have relatively higher prevalence of gastroschisis, more prevention work should be carried out in these areas to reduce the risk of gastroschisis.

## Material and Methods

### Study population and data source

Liaoning Women and Children’s Health Hospital is one of the sole obstetrical and gynecological hospitals for the province of Liaoning. It has also been a comprehensive care institution and has been in charge of the women’s and children’s health care guidance. Data from 2006 to 2015 were retrieved from the maternal and child health certificate registry of Liaoning Province which was maintained by this hospital. Hospital-delivered live birth and stillbirth infants were all included in this registry as the monitored subjects. This registry covers all 14 cities of the province (Shenyang, Dalian, Anshan, Fushun, Benxi, Dandong, Jinzhou, Yingkou, Fuxin, Liaoyang, Panjing, Tieling, Chaoyang, Huludao), with approximately 42 million inhabitants. The maximal diagnosis time for a congenital malformation case was the seventh day after birth^15^.

The details procedures of data collection were described in previous report^15^. Briefly, a ‘Birth Defects Register Form’ was used to collect the related information on the infants with gastroschisis. Once a gastroschisis case was identified and confirmed at the monitored hospital, the mother of the infant was interviewed by a trained obstetric or pediatric specialist in order to complete the aforementioned register form. Subsequently, the ‘Birth Defects Register Form’ was first submitted to the local maternal and child health facility and then to the provincial maternal and child health hospital, which is Liaoning Women and Children’s Health Hospital. The data of these cases were reviewed and confirmed by a group of state-level experts in medical genetics and pediatrics^15^.

Gastroschisis is defined as a congenital malformation characterized by visceral herniation usually through a right side abdominal wall defect to an intact umbilical cord and not covered by a membrane, and excludes a hypoplasia of the abdominal muscles, a skin-covered umbilical hernia, or an omphalocele (www.icbdsr.org). Infants with gastroschisis with or without other birth defects were included as cases. However, infants with gastroschisis and an abdominal wall disruption with a phenotype consistent with either an amniotic band sequence or limb body wall complex or a recognized single gene disorder or chromosomal abnormality, such as Down syndrome, were excluded from this study^15^. If a case was reported as both gastroschisis and omphalocele or eversion of viscera, then the monitored hospital was asked to confirm the data and collect relevant information again, such as photographs and detailed descriptions, which were finally ascertained by state-level medical geneticists. For suspected gastroschisis cases that were diagnosed through prenatal ultrasound scans, case ascertainment after termination or examination after the birth were requested. Therefore, seven hundred and forty-seven cases were identified. Additionally, the total number of live births in the study window was 3,248,954.

The data quality control was described in detail in previous literature^15^. In brief, according to the program manual to ensure high quality data, the disease diagnosis, data collection, data checking, and medical records were verified by the expert group at each level. In addition, an independent retrospective survey was organized by the experts to find deficiencies and inaccuracies in the data^15^.

### Statistical analysis

Gastroschisis prevalence were calculated for nine 1-year time intervals from 2006 to 2015. The annual percentage change for gastroschisis prevalence was used to quantify the time trends^24^,^25^,^26^,^27^. A regression line was fitted to the natural logarithm of the rates, weighted by the number of cases, i.e. y = α + βx + ε, where y = ln (rate) and x = calendar year, and then the APC was calculated as 100 × (e^*β*^ − 1). The 95% confidence interval (CI) of the APC was calculated by the methods for population-based cancer statistics recommended by the National Cancer Institute^28^. All analyses were conducted using SPSS for Windows (version 22, SPSS Inc, Chicago, IL, USA). All statistical tests were two-sided, and *P*-values less than 0.05 were considered statistically significant.

## Additional Information

**How to cite this article**: Li, N. *et al*. Decreasing prevalence and time trend of gastroschisis in 14 cities of Liaoning Province: 2006–2015. *Sci. Rep.* **6**, 33333; doi: 10.1038/srep33333 (2016).

## Figures and Tables

**Table 1:** The number of live births in each city in Liaoning province, 2006 to 2015.

City	Year										Overall
	2006	2007	2008	2009	2010	2011	2012	2013	2014	2015	
Liaoning Province	306734	341432	330414	321353	307826	304079	353108	321171	364400	298437	3248954
Shenyang	52256	61108	59196	59200	57521	58335	69721	67854	80997	65118	631306
Dalian	38744	46652	48309	47900	48774	50490	62324	58722	71178	57641	530734
Anshan	29270	31305	29647	27721	25184	25603	28790	25855	36171	20798	280344
Fushun	11661	12997	12314	12337	11638	11556	12942	12016	12845	10138	120444
Benxi	8620	9435	8759	8842	8696	8261	9440	8700	9857	7627	88237
Dandong	15710	15725	14836	14274	13894	14038	15895	15111	17718	14278	151479
Jinzhou	24293	24261	23149	22342	21255	20098	22559	20860	16137	16985	211939
Yingkou	16987	18924	19667	19070	17947	18484	21309	14224	21684	16515	184811
Fuxin	14158	14142	13353	13322	12370	11800	13050	9662	9121	11752	122730
Liaoyang	12888	15039	13754	13200	12331	11386	13296	11702	12747	9251	125594
Panjing	9887	9669	10134	9009	8800	8867	10362	9644	8276	9197	93845
Tieling	21263	20298	21456	19854	18421	16945	18938	14960	17389	15269	184793
Chaoyang	28669	30980	31168	30574	27837	27207	31236	29919	30646	26083	294319
Huludao	22328	30897	24672	23708	23158	21009	23246	21942	19634	17785	228379

**Table 2:** The prevalence of gastroschisis in each city in Liaoning province, 2006 to 2015 (per 10,000 births).

City	Year										Overall
	2006	2007	2008	2009	2010	2011	2012	2013	2014	2015	
Liaoning Province	6.87	3.98	3.27	2.37	1.98	2.20	1.87	1.81	1.32	0.80	2.30
Shenyang	5.74	6.87	4.05	4.56	1.74	2.57	2.73	1.47	2.22	0.46	3.14
Dalian	3.36	3.00	4.14	1.25	0.41	1.78	2.25	2.72	2.11	1.39	2.20
Anshan	2.39	3.19	1.69	0.72	0.40	2.73	0.35	1.93	0.28	0.48	1.43
Fushun	3.43	6.16	5.68	2.43	5.16	0.87	4.64	2.50	0.78	1.97	3.40
Benxi	5.80	3.18	2.28	1.13	5.75	1.21	0.00	1.15	2.03	0.00	2.27
Dandong	3.18	3.82	3.37	4.20	3.60	2.14	3.15	1.99	0.00	0.70	2.57
Jinzhou	1.23	1.65	2.59	1.34	2.35	2.49	0.89	0.96	1.86	0.59	1.60
Yingkou	0.59	3.17	2.54	2.10	0.56	1.08	1.41	0.00	0.00	0.61	1.24
Fuxin	2.12	5.66	2.25	0.00	0.00	2.54	3.07	2.07	0.00	0.85	1.96
Liaoyang	3.88	7.31	5.82	2.27	4.05	1.76	2.26	5.13	1.57	0.00	3.58
Panjing	2.02	3.10	1.97	1.11	2.27	1.13	1.93	0.00	2.42	0.00	1.60
Tieling	3.29	2.96	1.40	1.01	1.63	1.18	0.00	0.00	0.00	0.00	1.24
Chaoyang	3.49	2.91	3.21	3.60	2.87	3.68	0.96	1.00	0.65	0.77	2.31
Huludao	3.58	1.94	3.24	2.95	3.45	2.86	1.72	3.19	1.02	2.25	2.63

**Table 3:** Trends in gastroschisis prevalence in each city of Liaoning during 2006–2015.

City	2006		2015		PC† (%)	APC† (%)	*P* value	95% CI
	Case	Rate*	Case	Rate*				
Overall	103	6.87	24	0.80	−76.05	−12.63	<0.01	−16.18, −8.93
Shenyang	30	5.74	3	0.46	−91.98	−16.31	<0.01	−22.97, −9.06
Dalian	13	3.36	8	1.39	−58.64	−6.76	0.14	−15.56, 2.96
Anshan	7	2.39	1	0.48	−79.90	−11.04	0.16	−25.17, 5.75
Fushun	4	3.43	2	1.97	−42.49	−10.06	0.11	−21.32, 2.81
Benxi	5	5.80	0	0.00	−100.00	−11.13	0.20	−26.27, 7.12
Dandong	5	3.18	1	0.70	−77.99	−8.42	0.07	−16.69, 0.66
Jinzhou	3	1.23	1	0.59	−52.32	−3.82	0.50	−15.28, 9.18
Yingkou	1	0.59	1	0.61	2.86	−14.36	0.11	−29.11, 3.47
Fuxin	3	2.12	1	0.85	−59.84	−9.43	0.20	−22.39, 5.71
Liaoyang	5	3.88	0	0.00	−100.00	−9.61	0.14	−21.47, 4.05
Panjing	2	2.02	0	0.00	−100.00	−2.08	0.67	−11.73, 8.63
Tieling	7	3.29	0	0.00	−100.00	−20.23	0.03	−31.96, −6.47
Chaoyang	10	3.49	2	0.77	−78.02	−13.50	0.03	−23.45, −2.25
Huludao	8	3.58	4	2.25	−37.23	−4.02	0.29	−11.66, 4.29

APC, annual percent change; CI, confidence interval; PC, percent change.

^*^Gastroschisis prevalence were expressed as per 10,000 live births.

^†^Percent change and annual percent change between 2006 and 2015 was calculated by the gastroschisis prevalence.

**Table 4:** The relative contributions of decreasing trend of gastroschisis prevalence of each city in Liaoning province during 2006 to 2015.

City	β	Contribution rate (%)
Shenyang	−0.18	11.84
Dalian	−0.07	4.65
Anshan	−0.12	7.78
Fushun	−0.11	7.05
Benxi	−0.12	7.85
Dandong	−0.09	5.85
Jinzhou	−0.04	2.59
Yingkou	−0.16	10.31
Fuxin	−0.10	6.58
Liaoyang	−0.10	6.72
Panjing	−0.02	1.40
Tieling	−0.23	15.03
Chaoyang	−0.15	9.64
Huludao	−0.04	2.73
